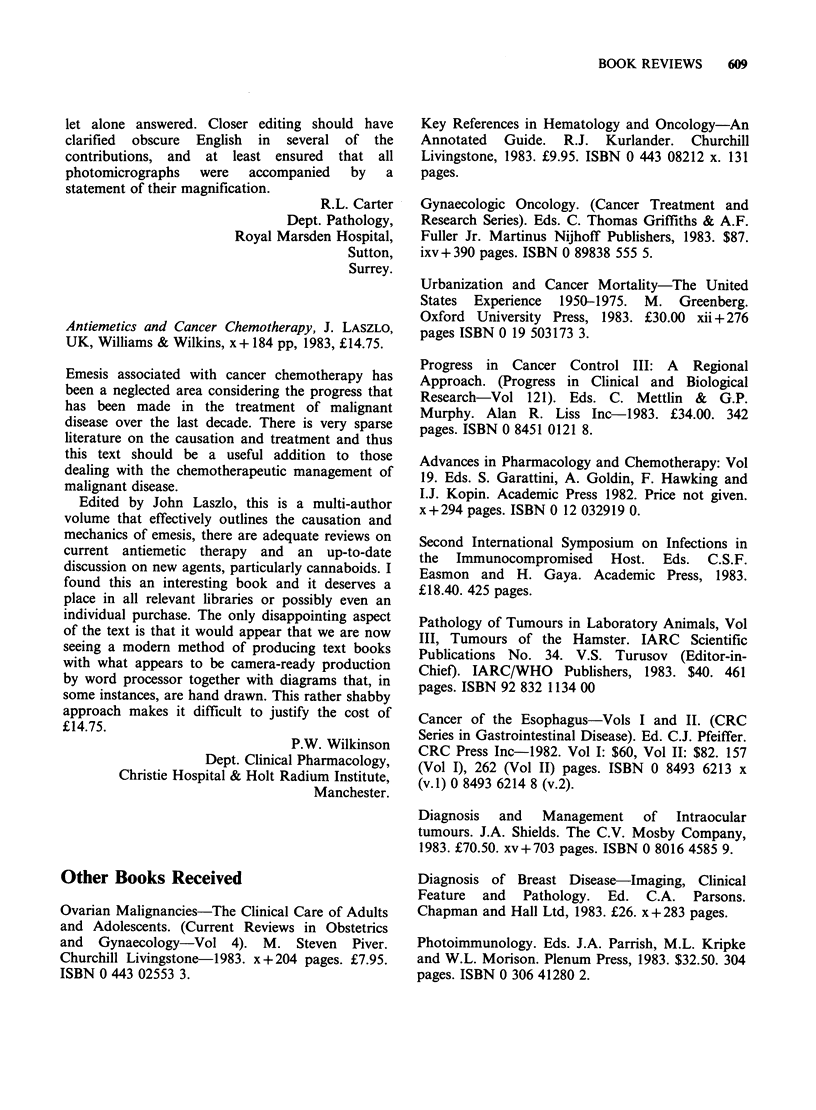# Antiemetics and Cancer Chemotherapy

**Published:** 1983-10

**Authors:** P.W. Wilkinson


*Antiemetics and Cancer Chemotherapy,* J. LASZLO, UK, Williams & Wilkins, x + 184 pp, 1983, £14.75.

Emesis associated with cancer chemotherapy has been a neglected area considering the progress that has been made in the treatment of malignant disease over the last decade. There is very sparse literature on the causation and treatment and thus this text should be a useful addition to those dealing with the chemotherapeutic management of malignant disease.

Edited by John Laszlo, this is a multi-author volume that effectively outlines the causation and mechanics of emesis, there are adequate reviews on current antiemetic therapy and an up-to-date discussion on new agents, particularly cannaboids. I found this an interesting book and it deserves a place in all relevant libraries or possibly even an individual purchase. The only disappointing aspect of the text is that it would appear that we are now seeing a modern method of producing text books with what appears to be camera-ready production by word processor together with diagrams that, in some instances, are hand drawn. This rather shabby approach makes it difficult to justify the cost of £14.75.

P.W. Wilkinson
Dept. Clinical Pharmacology,
Christie Hospital & Holt Radium Institute,
Manchester.